# Measuring cellular forces using bis-aliphatic hydrazone crosslinked stress-relaxing hydrogels†
†Electronic supplementary information (ESI) available. See DOI: 10.1039/c4sm01365d



**DOI:** 10.1039/c4sm01365d

**Published:** 2014-09-29

**Authors:** D. D. McKinnon, D. W. Domaille, T. E. Brown, K. A. Kyburz, E. Kiyotake, J. N. Cha, K. S. Anseth

**Affiliations:** a Department of Chemical and Biological Engineering , BioFrontiers Institute , University of Colorado , Boulder , Colorado 80303 , USA; b Howard Hughes Medical Institute , University of Colorado , Boulder , Colorado 80303 , USA

## Abstract

Studies focused on understanding the role of matrix biophysical signals on cells, especially those when cells are encapsulated in hydrogels that are locally remodelled, are often complicated by appropriate methods to measure differences between the bulk and local material properties. From this perspective, stress-relaxing materials that allow long-term culture of embedded cells provide an opportunity to elucidate aspects of this biophysical signalling. In particular, rheological characterization of the stress relaxation properties allows one to link a bulk material measurement to local aspects of cellular functions by quantifying the corresponding cellular forces that must be applied locally. Here, embryonic stem cell-derived motor neurons were encapsulated in a well-characterized covalently adaptable bis-aliphatic hydrazone crosslinked PEG hydrogel, and neurite outgrowth was observed over time. Using fundamental physical relationships describing classical mechanics and viscoelastic materials, we calculated the forces and energies involved in neurite extension, the results of which provide insight to the role of biophysical cues on this process.

## Abstract 2

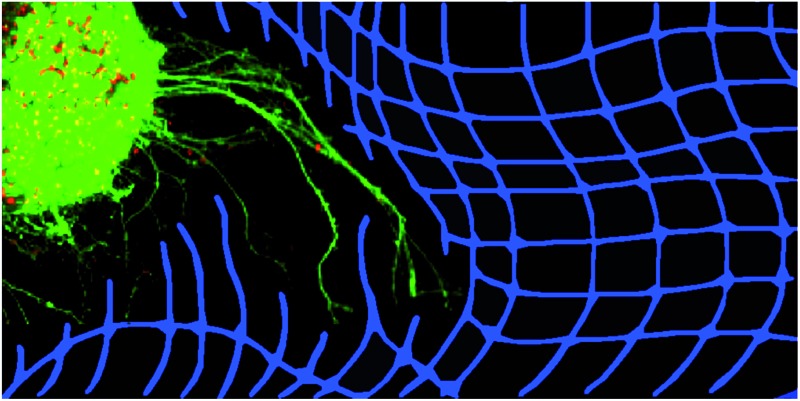
A covalently adaptable hydrazone-crosslinked PEG hydrogel is used to measure the force of extension exerted by motor neurites.

## Introduction

For the past decade, there has been a growing literature devoted to elucidating the effects of matrix modulus on the function of both plated and encapsulated cells.^1–8^ The Discher group pioneered some of the early efforts and demonstrated that human mesenchymal stem cells (hMSCs) preferentially commit to a lineage that is heavily dependent on the mechanics of the surface on which they are seeded.^2^ With corresponding trends observed for osteogenesis, myogenesis, and neurogenesis and even tissue formation, this finding provided quantitative evidence that altered many approaches to stem cell culture.^9^ Typically, stem cells are plated on polystyrene or glass dishes, both of which have a modulus that exceeds that of soft tissues by *ca.* six orders of magnitude. Discher's findings indicated that MSCs cultured on such materials were driven towards osteogenesis. These experiments were repeated with embryonic stem cells, yielding similar results.^10^ Thus, great interest arose in better understanding this mechanotransduction, especially in the context of soluble factors often added to media, on cell function.

More recently, many of these hypotheses have been extended to three-dimensional culture platforms.^8,11,12^ However, critical differences can arise between two- and three-dimensional culture systems. The *in vivo* morphology of adhesion dependent cells is typically spread and connected to neighbours through cadherins, tight junctions, and other interactions. This behaviour is enabled by the *in vivo* extracellular matrix, which is both porous and degradable, allowing cells to locally remodel or manoeuvre through the matrix. Synthetic hydrogels used for many of the original mechanotransduction studies did not allow either process, unless some mechanism of degradation – hydrolysis,^13,14^ light,^15,16^ or enzymatic degradation^17,18^ – was specifically engineered into the crosslinkers. Enzymatically degradable hydrogels have become increasingly popular, as these materials can be locally degraded by cells without *a priori* knowledge of the desired degradation rate, as is required for hydrolysis, photolysis, or some other external trigger. One disadvantage to this approach is that the rate of local degradation is unknown, and techniques to characterize the local modulus in 3D are complex. Efforts include traction force microscopy, which involves embedding fluorescent beads into the material and monitoring their displacement both in the presence and in the absence of cells. From the images collected and basic stress–strain calculations, it is possible to infer the amount of force encapsulated cells apply to the material.^8,19–21^ However, a critical assumption in traction force microscopy is that the local material modulus matches that of the bulk. This is likely not true in the context of the locally degradable gels that have been employed. Additionally, microrheology, using the Brownian motion of probe particles to infer material properties, has also been employed to investigate the local mechanics of hydrogel networks.^22,23^ This technique is restricted to very soft materials close to their gel point, which limits its broader applicability.

To address some of these complexities, we sought to design a biomaterial for cell culture, the bulk properties of which would closely mimic those found in the local cellular microenvironment. A reversibly crosslinked hydrogel that relaxes in response to stress applied by encapsulated cells or secreted ECM components should allow proper function without having to pre-program degradation rates, while maintaining constant modulus throughout the gel; this would enable accurate observations of the effect of matrix modulus on encapsulated cells. Previously, we reported that a PEG hydrogel material crosslinked with rapidly reversible bis-aliphatic hydrazone bonds results in a stress-relaxing, viscoelastic, cytocompatible hydrogel that allows the observation of cellular function in a highly controlled and characterized biophysical environment.^24,25^ Here, we demonstrate a simple method for making this material compatible with the culture of sensitive cell types and show how this material can be used to study the biophysical forces involved in neurite extension.

## Results & discussion

**Fig. 1:**
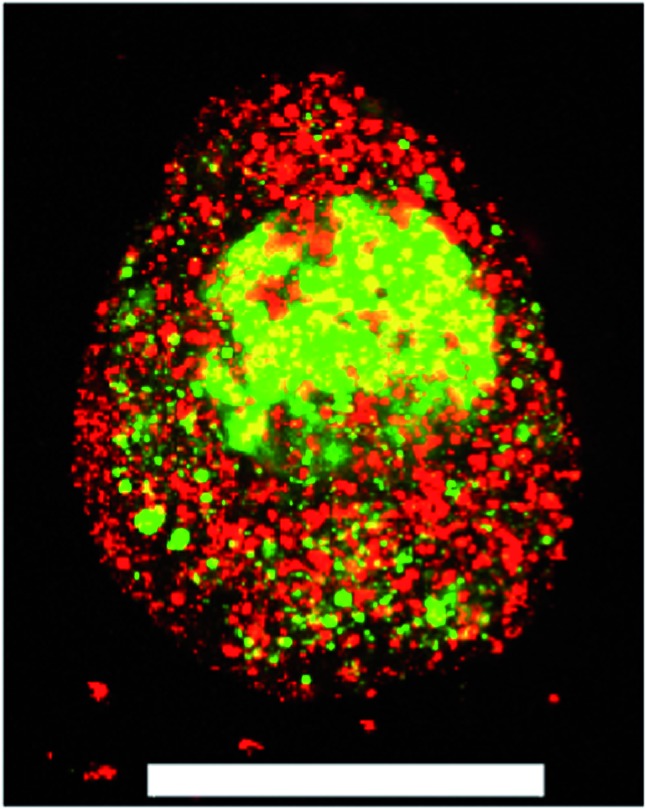
A ESMN embryoid body stained with calcein (green, live cells) and ethidium homodimer (red, dead cells) after encapsulation in a 1 : 1 PEG-hydrazine–PEG-aldehyde hydrogel. Scale bar is 200 μm.

Covalently adaptable networks were formed from PEG precursors to yield a hydrazone crosslinked hydrogel. The synthesis and characterization were previously described in detail.^24,25^ Briefly, 8-arm 10 kDa PEG macromers were functionalized with either aliphatic aldehyde or aliphatic hydrazine end groups and dissolved in buffered stock solutions. Upon preparing stoichiometrically balanced solutions, these formulations react in *ca*. 5 minutes to form a hydrogel with an equilibrium unswollen shear elastic modulus of *ca.* 30 kPa (Fig. ESI1†). Due to the rapid reversibility of the bis-aliphatic hydrazone bond, these materials are able to relax applied stress on the order of minutes.^25^ An RGDS peptide coupled to the benzaldehyde moiety was used to confer adhesive functionality to the material for culturing primary ES derived motor neurons. This material was previously shown to be highly compatible for the long-term culture of C2C12 myoblasts;^24^ however, initial attempts to encapsulate and culture primary motor neurons results in nearly uniform cell death at the material–embryoid body interface (Fig. 1).

To identify the precise component and concentrations that negatively affect cell viability, we examined the effect of 10 mM non-gelling solutions of PEG-aldehyde, PEG-hydrazine, and PEG-hydrazone on the viability of a more adherent primary cell culture. Neither the PEG-hydrazone nor the PEG-hydrazine had a statistically significant impact on cell viability or ATP content in adherent cultures. In contrast, treating plated cells with a 10 mM solution of PEG-aldehyde resulted in substantial cell death within hours (Fig. ESI2†). These results are in line with previous reports that examined the toxic effects of aldehydes on cell viability and their possible contribution to neurodegenerative diseases.^26^ Taken together, our results and the well-established negative influence of aldehydes on cell health strongly suggest that minimizing the amount of reactive aldehyde in the gel formulations should lead to increased cell viability.

**Fig. 2:**
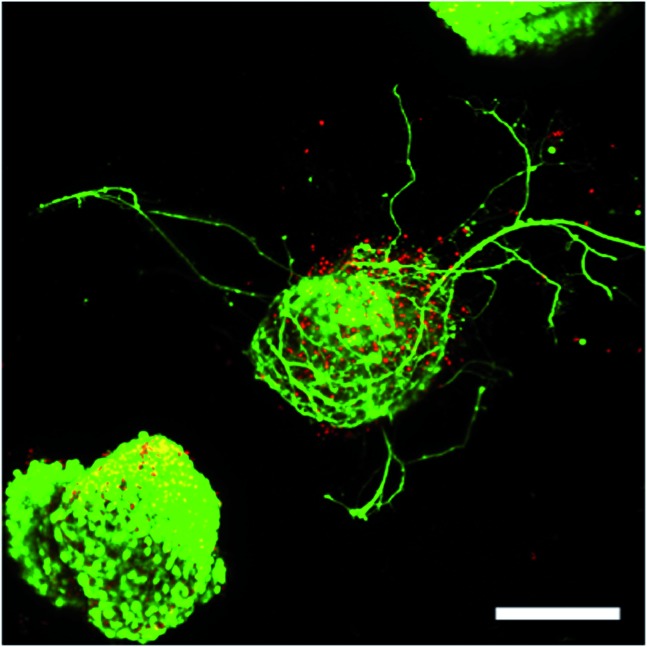
ESMN embryoid bodies stained with Calcein (green), indicating live cells, and Ethidium Homodimer (red), indicating dead cells, after two days in culture. The gel formulation was 80 mM PEG-hydrazine with 40 mM PEG-aldehyde, in contrast to the 80 mM of both used for the gel formed in Fig. 1. High cell viability was observed and many embryoid bodies were observed to extend axons throughout the material. Such extension is possible only in gels with a stress relaxation-mediated mechanism. Scale bar is 200 μm.

Because free PEG-hydrazine–PEG-aldehyde and the resulting hydrazone are in rapid equilibrium, we reasoned that we could shift the equilibrium toward the PEG-hydrazone – a component that had no significant effect on cell survival – by increasing the amount of PEG-hydrazine in the gel formulation to minimize the concentration of “free” PEG-aldehyde. Empirically, we observed that hydrogels polymerized with a 2 : 1 stoichiometry of PEG-hydrazine–PEG-aldehyde resulted in a 3-dimensional cell culture scaffold that showed much lower toxicity to ESMN embryoid bodies as determined by a live/dead assay. Not only did the encapsulated ESMN embryoid bodies show dramatically improved viability, but we also observed neurite extension from many embryoid bodies (Fig. 2).

Having identified a hydrazone gel formulation that was compatible with ESMN viability and neurite outgrowth, we next sought to characterize the mechanical properties of the hydrogel formed at the 2 : 1 stoichiometric ratio of hydrazine to aldehyde functional groups. To this end, macromer solutions were pipetted onto a parallel plate rheometer, and the shear storage and loss moduli were recorded. The elastic equilibrium modulus and time constant for gelation were quantified by fitting the data to an exponential model, where *G*_eq_ is the equilibrium unswollen shear elastic modulus, *A* is a fitting parameter, *τ* is the time constant of evolution, and *t* is the time (eqn (1)).

1 *G*(*t*) = *G*_eq_ – *A*e^–*tτ*^

**Fig. 3:**
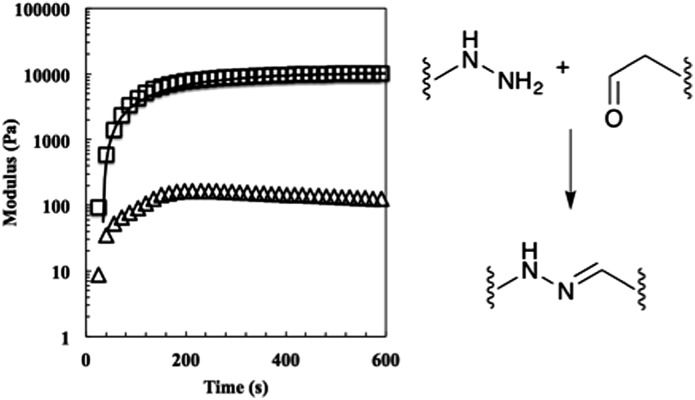
The evolution of the 50% off-stoichiometry bis-aliphatic hydrazone crosslinked hydrogel. The squares represent the shear storage modulus, the triangles the shear loss modulus, and the solid line an exponential fit that was used to determine the equilibrium modulus, *G*_eq_, and the time constant of evolution, *τ*. The hydrogel reached a 90% of its *G*_eq_ of 11 000 ± 700 Pa in 410 ± 30 s. The bis-aliphatic hydrazone crosslinking reaction is show at right.

Using these fits, the 2 : 1 gels reached a mean unswollen modulus of 11 000 ± 700 Pa in 410 ± 30 s. As expected, this value is much lower than that of the stoichiometric gel (Fig. 3).^25^

**Fig. 4:**
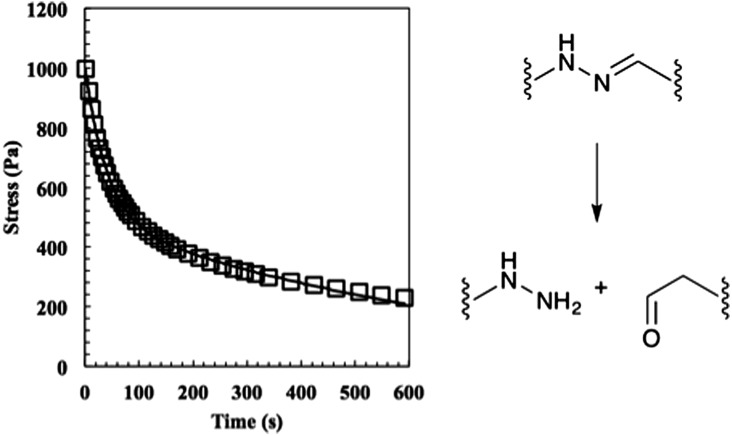
The relaxation of the 50% off-stoichiometry bis-aliphatic hydrazone crosslinked hydrogel. The squares represent the stress and the solid line an exponential fit that was used to determine the equilibrium modulus the time constants of evolution, *λ*_slow_ and *λ*_fast_. The hydrogel relaxed with a *λ*_slow_ of 690 ± 50 s and a *λ*_fast_ of 38 ± 3 s. The hydrolysis of the bis-aliphatic hydrazone bond, the rate of which was previously determined to govern relaxation rates,^25^ is shown at right.

Next, the relaxation characteristics of the gel were explored. We expected the 2 : 1 gel to relax differently than a stoichiometrically balanced gel, but were unsure whether the characteristics would be dominated by the unreacted end groups or the shifted equilibrium. In theory, the former should speed the rate of relaxation and the latter should slow it. Hydrogels were allowed to equilibrate for 30 min on the rheometer, strained 10%, and stress measurements were recorded (Fig. 4). The data were initially fit to eqn (2) where *σ*_0_ is the initial stress, *λ* is the time constant of relaxation, and *t* is the time, which was previously shown to fit the relaxation of the stoichiometric bis-aliphatic hydrazone gels.

2 *σ*(*t*) = *σ*_0_e^–*t*/*λ*^

 Eqn (2) is derived from the stress relaxation of a single spring in series with a single dashpot and describes ideal Maxwellian viscoelastic fluids. However, the rheological data for this 2 : 1 system did not fit the model. Rather, it was clear that the material relaxed with two distinct modes, a fast mode and slow mode. Quantitatively, the behaviour could be described by eqn (3), which physically represents a spring series with a fast dashpot in parallel with a spring in series with a slow dashpot.

3 *σ*(*t*) = *σ*_0_(e^–*t*/*λ*_fast_^ + e^–*t*/*λ*_slow_^)

Using this analysis, we found *λ*_slow_ = 690 ± 50 s and *λ*_fast_ = 38 ± 3 s, indicating that the shifted equilibrium constant and the unreacted functional groups both contribute to the 2 : 1 hydrogel relaxation properties. We hypothesize that the initial rapid rate of relaxation is due to the unreacted ends physically dragging through the network disentangling crosslinks and that the slow rate of relaxation is due to the hydrazone bond reversing.

We next sought to exploit the well-defined and predictable mechanical properties of these reversibly crosslinked gels to measure the force that an axon must exert to extend from its nascent to final position. Basic mechanics dictates that the energy (*E*) required to travel through a viscoelastic fluid is the path integral of the stress function (*σ*(*t*)) times the surface area (*A*) (eqn (4)).4
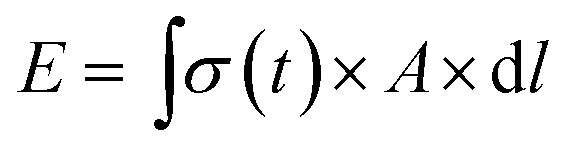



Furthermore, the power (*P*) exerted during axon extension and the average force (*F*) over the same period are calculated using eqn (5) and (6), where *l* is the path that is integrated over to calculate the energy in eqn (4).5
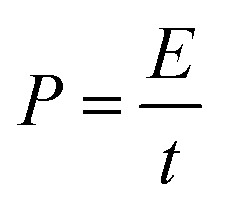

6
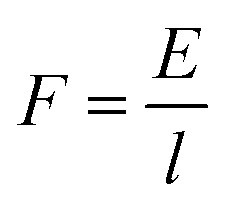



Thus, if the stress function for the material were known and the position of the growth cone could be tracked over time, many fundamental biophysical properties of the cells can be readily calculated from these first principles. Exploiting our rheological characterization of the gel formulations (Fig. 4, eqn (3)), we were able to develop a general equation for the energy required for a process to navigate the material.

Substituting eqn (3) function into eqn (4) renders a path integral over an expression with a time dependence (eqn (7)).7
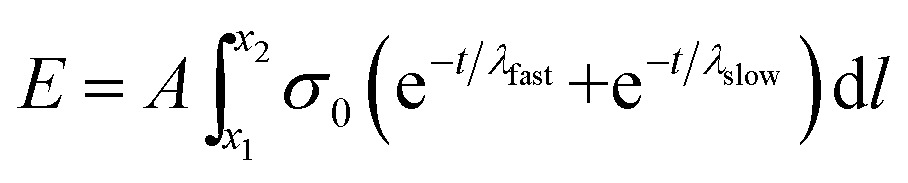



However, if the speed of axon extension is relatively constant throughout the experiment (*i.e.*, constant between time steps), then time can be exchanged for position (eqn (8)).8
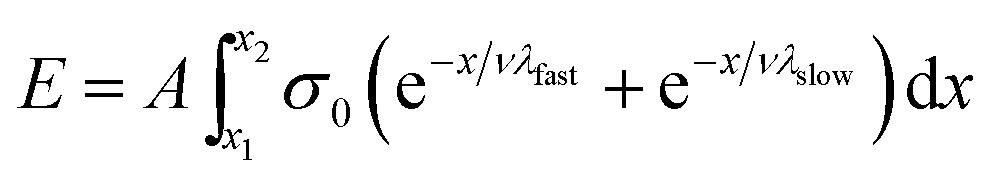
here, *v* is the mean velocity of the axon growth cone over the time interval. Integration of eqn (8) yields the energy required to travel from point *x*_1_ – set to 0 here for brevity, but the general equation is easily derived – to point *x*_2_ through any viscoelastic fluid given a constant velocity over that period (eqn (9)).

9 *E* = *Aνσ*_0_(*λ*_fast_ + *λ*_slow_ – *λ*_fast_e^–*x*_2_/*λ*_fast_*ν*^ – *λ*_slow_e^–*x*_2_/*λ*_slow_*ν*^)

**Fig. 5:**
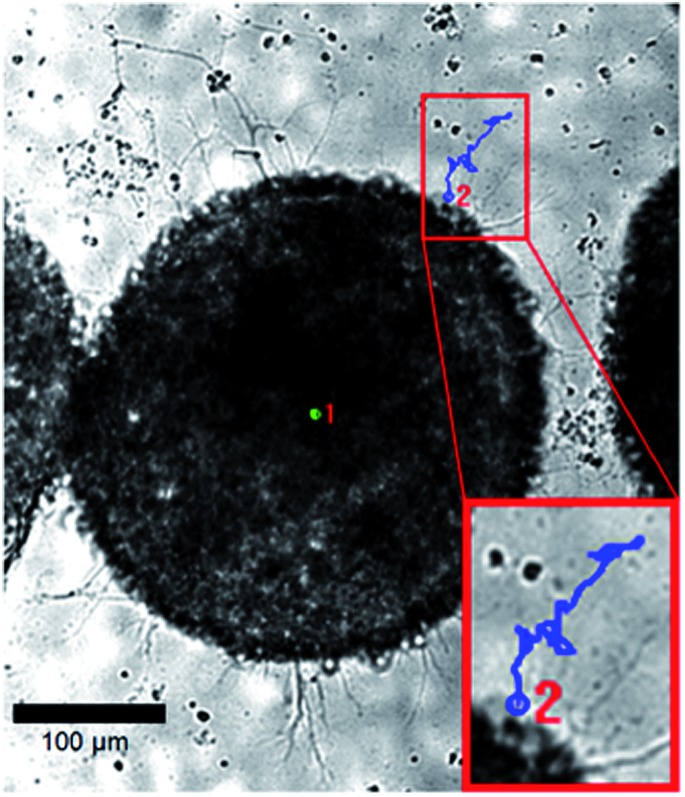
Example brightfield images of an ESMN embryoid body encapsulated in the hydrazone-crosslinked hydrogel. The blue line is the path of an axon extending over 24 hours and the green dot indicates the extent of sample drift. Scale bar is 100 μm.

To apply this equation to the analysis of motor axons extending through viscoelastic hydrazone gels, we encapsulated ESMN embryoid bodies in a 2 : 1 stoichiometry formulation and monitored axon extension for 48 hours, taking an image every five minutes (Fig. 5). The position of the tip of the neurite was noted at each frame for five neurites in three different hydrogels.

**Fig. 6:**
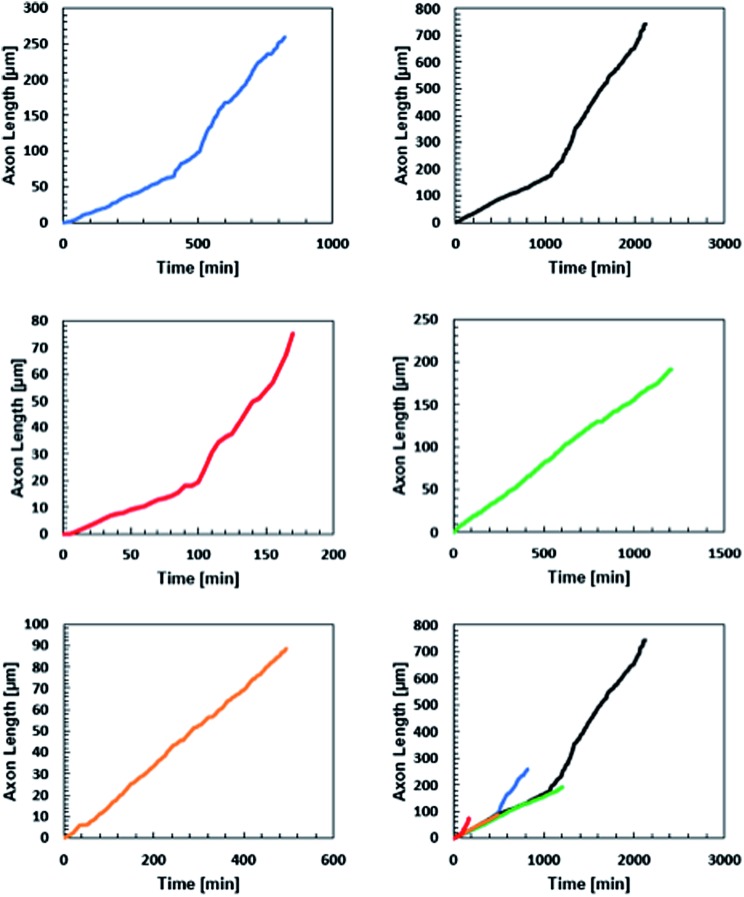
Axon displacement as a function of time for five axons extending from five separate embryoid bodies in three different gels. Initial rates of axon extension were 8.2 ± 1.5 μm per hour. The change in slope of the black, blue, and red traces were likely due to the axon reaching the surface of the gel.

All five neurites followed a remarkably similar trajectory at early time points, pushing through the gel at 8.2 ± 1.5 μm hour^–1^ (Fig. 6). Three of the neurites dramatically increased their speed to 27 ± 7 μm hour^–1^. A careful examination of the microscopy images reveals that this velocity change is likely a result of the axons reaching the surface of the gel and switching from an extension that is mechanically inhibited by the gel to simply extending along the surface.

**Fig. 7:**
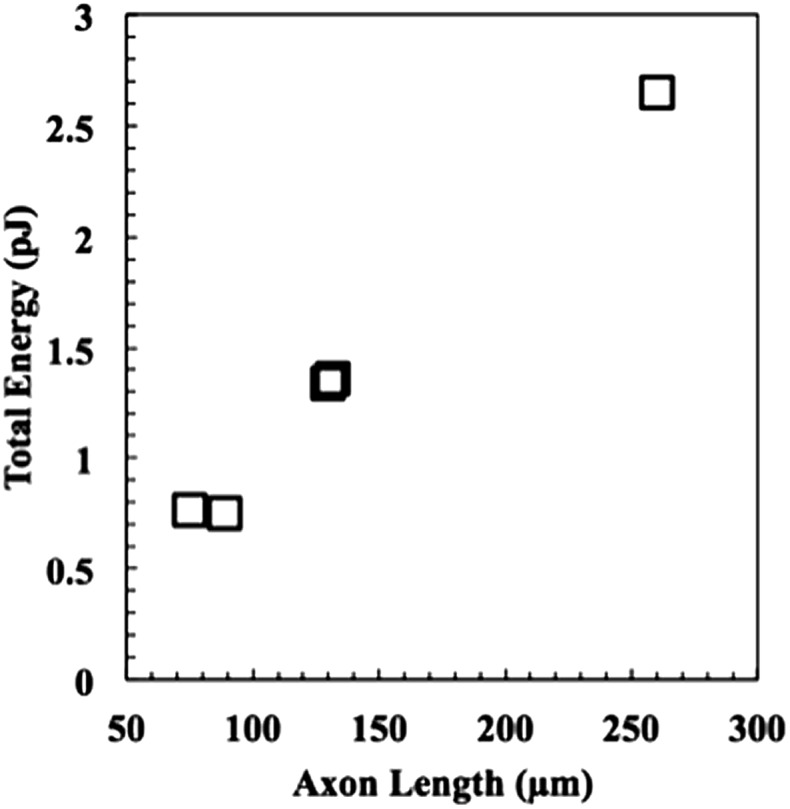
The energy expended to project the neurite through the material as a function of length. To put these numbers into a more familiar biological context, the energy required to extend a neurite through the material ranges from *ca*. 1/3 to 1 femtomole of ATP, assuming each mole yields 30 kJ.

Next, the energies of extension were calculated using eqn (9), and the results are plotted *versus* axon length in Fig. 7. Neurite length and energy appeared linearly correlated, as one might expect based on the results in Fig. 6, which show that all neurites travel approximately the same speed through the material. Using these data, an average force of 9.9 ± 0.8 nN for the five neurites was calculated using eqn (4) and (6), which was for the five neurites analysed. These values are consistent with values reported for cellular forces using traction force microscopy and other methods. For example, fibroblasts seeded on a surface of a bed of microneedles were shown to exert 1–20 nN of traction force.^27,28^ Fibroblasts seeded on the surface of a hydrogel embedded with fluorescence beads were shown to exert 50–500 Pa of traction force, which corresponds to 5 pN to 50 nN, assuming the stress acts over an area of 10 μm^2^.^21^ Axons extending from PC12 cells, a neuronal cell line, were deflected mechanically and forces of adhesion were measured to be in the 1–5 nN range.^29^ Chick sensory neurons were shown to advance with similar forces.^30,31^ While all these experiments were performed with cells plated on a surface, rather than encapsulated in a hydrogel, the reported values for cellular forces are very similar to those reported here. This is likely the first time the force of an extending motor neurite has been measured by any method and work is in progress to validate these results through other methods. In general, we believe that these covalently adaptable hydrogels, with their reversible crosslinks, may prove as a useful system for culture of primary cells. The viscoelastic properties capture many aspects of the biomechanics of the native extracellular matrix, while allowing cells to proliferate, extend processes and deposit matrix molecules. Since the gel mechanical properties can be readily manipulated in a systematic way, these materials should prove useful in efforts to better characterize the complex exchange of biophysical cues between cells and their microenvironment.

## Experimental

Unless otherwise noted, all chemicals and solvents were of analytical grade and used as received from commercial sources. 8-arm 10 kDa PEG-amine and 8-arm 10 kDa PEG-alcohol were obtained from JenKem. Water (dd-H2O) used in biological procedures or as a reaction solvent was deionized using a central system. Peptide synthesis was carried out on a Tribute Peptide Synthesizer (Protein Technologies, USA). Confocal images were taken on a Zeiss LSM 710 (Carl Zeiss Microscopy, Germany).

### Macromer synthesis

10 kDa PEG-octa-hydrazine (8-H) was synthesized by dissolving tri-Boc-hydrazinoacetic acid (1.372 g, 3.52 mmol, 2.1 equiv. per amine) in anhydrous DMF (10 mL) and was activated with HATU (1.216 g, 3.20 mmol, 2.0 equiv.) and *N*-methylmorpholine (0.792 mL, 7.2 mmol, 4.5 equiv.). The reaction was stirred for 5 minutes, and then the 4-arm 20 kDa (8.000 g, 0.4 mmol) PEG was added, and the reaction was allowed to proceed overnight at room temperature. The product was precipitated in ice-cold diethyl ether, dried, treated with a solution of 50 : 50 DCM–TFA for 4 hours to remove the Boc group. The resulting compound was precipitated in ether, dissolved in DI water, dialyzed (2 000 MWCO) against DI water for 24 hours, and lyophilized, after which it was used for experimentation. 1.25 kDa PEG-mono-hydrazine, used for the toxicity experiments, was synthesized in an identical manner except the 2 000 MWCO dialysis tube was replaced with a 500 Da MWCO tube.

8-H ^1^H NMR (D_2_O, 400 MHz): *δ* = 3.59 (s, PEG).

8-H ^1^H NMR(DMSO-d_6_, 400 MHz): *δ* = 8.1 (t, *J* = 4 Hz, 1H), *δ* = 4.45 (m, 2H), *δ* = 3.51 (s, PEG), *δ* = 3.2 (m, 1H), *δ* = 2.96 (m, 2H).

10 kDa PEG-octa-aldehyde (8-AA) was synthesized using a Swern oxidation.^32^ Oxalyl chloride (1.5 mL, 17.6 mmol, 11 equiv. per hydroxyl) was dissolved in anhydrous DCM (20 mL) in a flame-dried flask purged with argon, and the reaction flask was cooled in an acetone/dry ice bath. DMSO (1.3 mL, 18.5 mmol, 11.5 equiv.) diluted 1 : 5 in anhydrous DCM was added dropwise over the course of 5 minutes. The reaction was allowed to proceed for 10 minutes to ensure formation of the alkoxysulfonium ion intermediate. 8-arm 10 kDa PEG-OH (2 g, 0.2 mmol) or 4-arm 10 kDa PEG-OH (4 g, 0.4 mmol) was dissolved in anhydrous DCM (5 mL) and added dropwise over 10 minutes and allowed to react for 2 hours. Triethylamine (5.6 mL, 40 mmol, 25 equiv.) was added dropwise over 10 minutes and given 20 minutes to react. Finally, the reaction was allowed to warm to room temperature, and the product was precipitated in ether and dialyzed as previously described.

8-AA ^1^H NMR (D_2_O, 400 MHz): *δ* = 5.04 (t, *J* = 6 Hz, 1H), *δ* = 3.76 (t, *J* = 4 Hz, 2H), *δ* = 3.59 (s, PEG). Aldehyde exists in the diol form in D2O.

8-AA ^1^H NMR (DMSO-d_6_, 400 MHz): *δ* = 9.61 (s, 1H), *δ* = 4.23 (s, 2H), *δ* = 3.54 (s, PEG). 8-AA gels in organic solvents; therefore, NMR peaks were significantly broadened.

### Rheology

Samples were formed *in situ* by pipetting 30 μL monomer solutions between the bottom Peltier plate and a flat tool 8 mm in diameter on a shear rheometer. The gap was closed to 500 μm as quickly as possible, and the experiment was commenced. Evolution experiments were performed at 25 °C and relaxation experiments were performed at 37 °C. Frequency and strain sweeps were performed to ensure measurements were made in the linear region. Evolution experiments were performed at 1% strain and 1 rad s^–1^; frequency sweeps were performed at 1% strain; and stress relaxation experiments were performed at 10% strain. All experiments were performed in triplicate with the error representing the standard deviation.

### Cell encapsulation

Gels were prepared with a total volume of 30 μL from stock solutions of 8-H, 8-AA, and benzaldehyde-KGRGDS, the pH of which had been adjusted to 7.4. Peptide, cells, and 8-H were gently mixed prior to the addition of the 8-AA after which the solution was triturated 10 times and pipetted into a mould. After 3 minutes, the 8-H : 8-AA gels were placed in pre-warmed media. Final concentrations of aldehyde, hydrazine, and KGRDGS functional groups were 24 mM, 48 mM, and 1 mM, respectively.

### Toxicity

hMSCs were plated and allowed to spread and adhere for 48 hours. Cells were then treated with 10 mM functional groups of 10 kDa 8-arm PEG-alcohol, 8-AA, 1.25 kDa PEG-mono-hydrazine (1-H), or both 8-AA and 1-H for two hours. Cells were then rinsed and ATP content was measured using the CellTiter-Glo Luminescent Cell Viability Assay.

### ES cell culture

ES cells were differentiated into spinal motor neurons as previously described.^33,34^ Briefly, Hb9::GFP mouse embryonic stem cells were plated into ES cell medium (ES DMEM, ES FBS, glutamine, non-essential amino acids, nucleosides, 2-mercaptoethanol, LIF (Life Technologies)) at approximately 5 × 10^5^ cells per gelatinized T25 flask. After 24 hours the media was replaced, and on day 2 of culture, ES cells were trypsinized and placed in suspension culture in motor neuron media (Advanced-DMEM/F12, Neurobasal, and Knockout Serum Replacement (Life Technologies)) at 5 × 10^5^ cells per untreated 10 cm tissue culture dish. In suspension culture, the cells aggregated into embryoid bodies (EBs). Two days after initial seeding the EBs were split 1 : 4 and induced into motor neurons with 1 μM retinoic acid (RA) (Sigma) and 1 mM smoothened agonist (SAG) (Millipore). After 3 days of exposure to RA and SAG, the EBs displayed strong expression of Hb9 : GFP transgene.

### Staining

For Live/Dead imaging, ESMNs lacking the Hb9::GFP transgene were used. Gels were incubated in a 2 μM calcein AM and 4 μM ethidium homodimer-1 solution for 30 minutes on a shaker in a cell culture incubator and then imaged.

### Confocal imaging

Gels were placed between a glass slide and a coverslip separated by a rubber gasket and were imaged using a 10×, 20× or 40× water immersion objective. A 488 nm laser was used to excite eGFP, calcein AM, and ethidium homodimer. For Live/Dead imaging a Z-stack of 100 images was taken through the first 600 μm of the gel in three different fields of view with 7.75 μm between images.

### Real-time imaging

Cell motility in cell-laden hydrogels was characterized using a Nikon TE 2000-E microscope with a Nikon environmental chamber and an external heater (*In vivo* Scientific) and CO2 regulator (*In vivo* Scientific). Hydrogels were polymerized and swollen as described previously, then placed in a 24-well culture insert plate (BD Falcon, Fisher) and held in place by a transwell insert (Becton Dickinson) with the bottom removed by a 5 mm biopsy punch. Fresh experimental medium was placed in the well at the beginning of each experiment. Real-time tracking was performed using Metamorph software for automated stage control, image collecting and positional cell tracking. After 2 h in culture, brightfield images of axonal growth were taken every 5 min for 24 h. Using Metamorph, manual *x*–*y* positions of the growth cone were tracked at each time point to determine axonal growth.

## Conclusions

Here, we have shown that the combination of well-defined viscoelastic materials, fundamental physics, and real-time microscopy allows for the measurement of basic biophysical processes that are extremely difficult to measure by other means. The encapsulation of ESMN embryoid bodies in well-characterized bis-aliphatic hydrazone crosslinked hydrogels has enabled the determination, for the first time, of the forces involved in motor neurite extension. However, in principle this technique could be extended to study any dynamic cellular process.

We have described a covalently adaptable hydrogel formulation that is compatible with primary cell culture. The viscoelastic properties capture many aspects of the biomechanics of the native extracellular matrix, while allowing cells to proliferate, extend processes, and deposit matrix molecules. Combining rheological measurements with basic mechanics equations allows us to derive a set of equations that, when coupled with time-lapse imaging of axon extension, enables the measurement of cellular forces. Because the gel mechanical properties can be systematically manipulated, these materials should prove useful in efforts to better characterize the complex exchange of biophysical cues between cells and their microenvironment.
