# Supplementary material for: Measuring cellular forces using bis-aliphatic hydrazone crosslinked stress-relaxing hydrogels
Source: Soft Matter. 2014 Sep 29;10(46):9230–6. doi: 10.1039/c4sm01365d (PMC4445372; doi:10.1039/c4sm01365d)
Supplement: Supplementary file 1 [file SM-010-C4SM01365D-s001.pdf]

Electronic Supplementary Material (ESI) for Soft Matter.
This journal is © The Royal Society of Chemistry 2014

Supporting Information

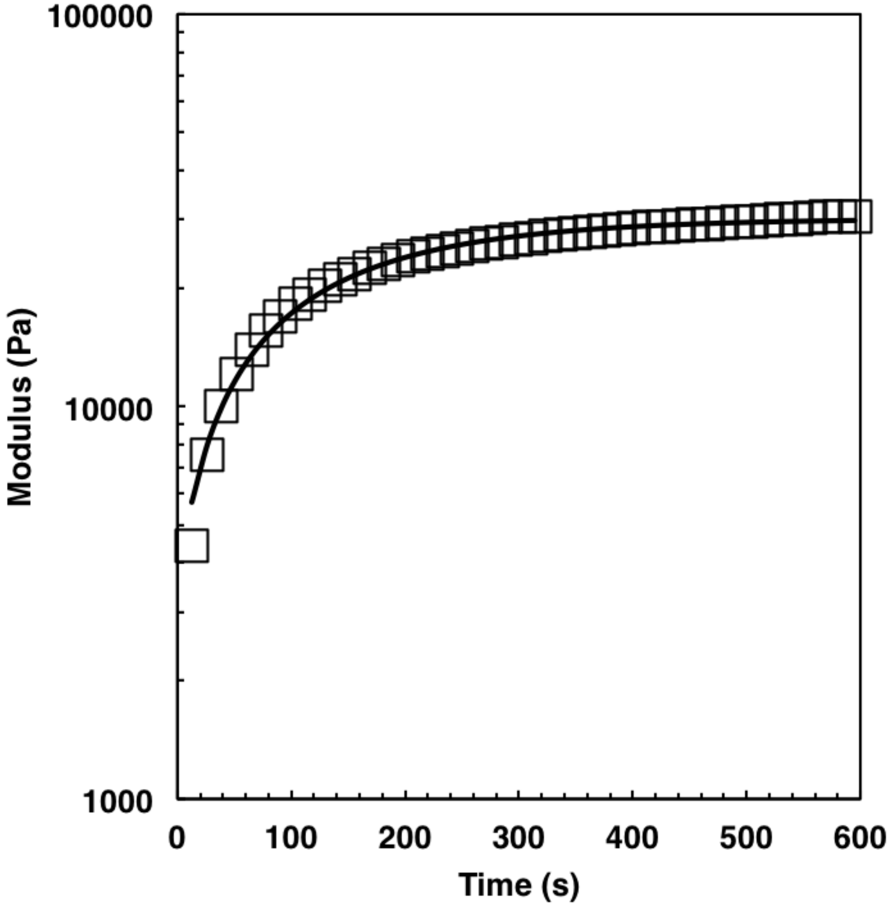

Figure 1. Evolution of the shear elastic modulus of the stoichiometric 8-H:8-AA hydrogel. Fitting these data to Equation 1, the equilibrium modulus, G_{eq}, is 30 kPa unswollen. After swelling, this value declines to *ca.* 10 kPa, which was previously reported. The data are the open squares and the fit is the solid line.

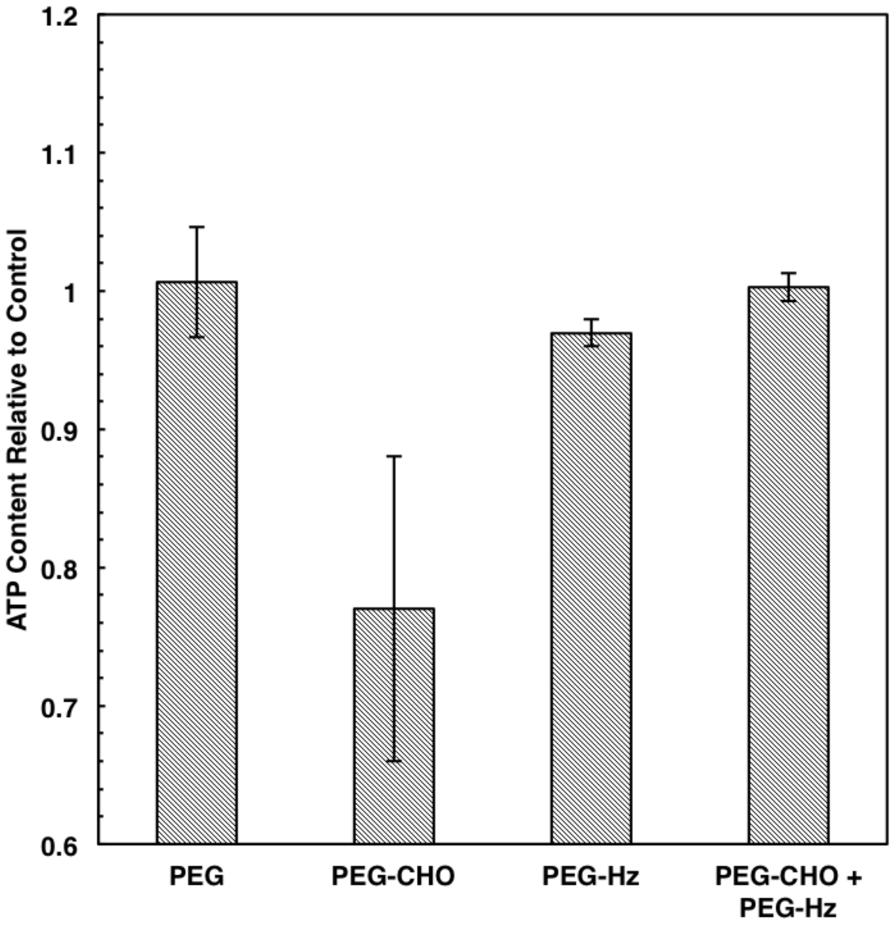

Figure 2. Plated human mesenchymal stem cells (hMSCs) were plated and allowed to adhere and spread for 48 hours. Cells were then treated with 10 kDa 8-arm PEG-alcohol, 10 kDa 8-arm PEG-aldehyde, 1.25 kDa monofunctional PEG-hydrazine, or a combination of both the aldehyde- and hydrazone-terminated PEG in concentrations 4.7 times lower than they were exposed to in the hydrogel (10 mM). After 2 hours of incubation, the cells were lysed and ATP content was measured. The ATP content of the PEG-aldehyde exposed cells was lower than the other three conditions, indicating PEG-aldehyde is deleterious to cell health. PEG-hydrazine mitigates this effect due to the hydrazine groups outcompeting the cellular functional groups for the free aldehydes. The error bars represent the standard deviation between three independent trials.
